# Supplementary material for: Directional dark field for nanoscale full-field transmission X-ray microscopy
Source: Light Sci Appl. 2026 May 8;15:223. doi: 10.1038/s41377-026-02263-z (PMC13153260; doi:10.1038/s41377-026-02263-z)
Supplement: Supplementary file 1 — Supplementary Information for Directional Dark Field for Nanoscale Full-Field Transmission X-Ray Microscopy [file 41377_2026_2263_MOESM1_ESM.pdf]

Supplementary Information for Directional Dark Field for Nanoscale Full-Field Transmission X-Ray Microscopy

SAMI WIRTENSOHN[1,2*], SILJA FLENNER[1], DOMINIK JOHN[1,2], PENG QI[3,4], CHRISTIAN DAVID[3], MANFRED MAY[5,6], PATRICK HUBER[5,6], DIRK HERZOG[7,8], STEFAN TANGL[9,10], CARINA KAMPLEITNER[9,10,11], KRITIKA SINGH[1], INGOMAR KELBASSA[7,8], KATRIN BEKES[12], JULIA HERZEN[2], IMKE GREVING[1]

[1] *Institute of Materials Physics, Helmholtz-Zentrum Hereon, Max-Planck-Straße 1, 21502 Geesthacht, Germany*
[2] *Research Group Biomedical Imaging Physics, Department of Physics, TUM School of Natural Sciences & Munich Institute of Biomedical Engineering, Technical University of Munich, James-Franck-Straße 1, 85748 Garching, Germany*
[3] *Center for Photon Science, Paul Scherrer Institut, Forschungsstrasse 111, 5232 Villigen, Switzerland*
[4] *Center for Transformative Science, ShanghaiTech University, Middle Huaxia Road 393, 201210 Shanghai, China*
[5] *Institute for Materials and X-Ray Physics, Hamburg University of Technology, Denickestr. 10, 21073 Hamburg, Germany*
[6] *Center for X-Ray and Nano Science CXNS, Deutsches Elektronen-Synchrotron DESY, Notkestr. 85, 22607 Hamburg, Germany*
[7] *Institute for Industrialization of Smart Materials, Hamburg University of Technology, Harburger Schloßstraße 28, 21079 Hamburg, Germany*
[8] *Fraunhofer IAPT, Am Schleusengraben 14, 21029 Hamburg, Germany*
[9] *Core Facility Hard Tissue and Biomaterial Research, Karl Donath Laboratory, University Clinic of Dentistry, Medical University of Vienna, Sensengasse 2a, 1090 Vienna, Austria*
[10] *Austrian Cluster for Tissue Regeneration, Donaueschingenstr. 13, 1200 Vienna, Austria*
[11] *Ludwig Boltzmann Institute for Traumatology, The Research Center in Cooperation with AUVA, Donaueschingenstr. 13, 1200 Vienna, Austria*
[12] *Department of Paediatric Dentistry, University Clinic of Dentistry, Medical University of Vienna, Sensengasse 2a, 1090 Vienna, Austria*
[*] *sami.wirtensohn@hereon.de*

To gain the difference image in Figure 2 B, the dark-field projection shown in Figure S1 A is subtracted from the extended dark-field projection in Figure S1 B. Due to the high (dynamic) range of the dark-field intensity values, the change is barely visible in the direct comparison.

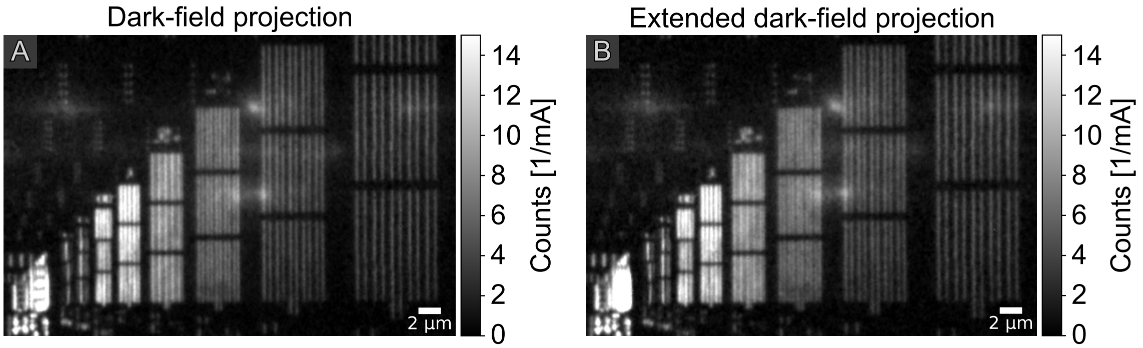

Fig. S1. Dark-field projection of a line pair test pattern with the left CAP closed (A). Due to the elongated shadow in the back focal plane of the FZP, the right DF-AP can be further opened to accept a larger maximum magnitude of the scattering vector, increasing the intensity, especially for smaller feature sizes (B). The difference of these dark-field projections is used in Figure 2. Each of the dark-field projections is exposed for a total of 200 s.
